# Anthropogenic microparticles and mercury co-occurrence in blue sharks from the Tropical Eastern Pacific

**DOI:** 10.1007/s11356-026-38018-x

**Published:** 2026-07-04

**Authors:** Maria Emilia Rechimont, Jorge Ruelas-Inzunza, Victor Jesus Núñez-Flores, Felipe Amezcua

**Affiliations:** 1 Instituto de Ciencias del Mar y Limnología, Universidad Nacional Autónoma de México, Joel Montes Camarena S/N, 82040 Mazatlán, Sinaloa Mexico; 2 Instituto Tecnológico de Mazatlán, Calle Corsario 1 No. 203, 82070 Mazatlán, Sinaloa Mexico; 3 Facultad de Ciencias del Mar. Paseo Claussen S/N, Universidad Autónoma de Sinaloa, Col. Los Pinos, CP 82000 Mazatlán, Mexico

**Keywords:** Non-plastic anthropogenic microparticles, Mercury, *Prionace glauca*, Tropical Eastern Pacific, Generalized additive models, Contaminant co-occurrence

## Abstract

Anthropogenic microparticles derived from synthetic polymers and industrially modified natural materials have become persistent pollutants in marine ecosystems because of their capacity to adsorb and transport other contaminants. In this study, we quantified anthropogenic microparticles (AMPs) ingestion and evaluated its relationship with total mercury concentrations (THg; liver + muscle) in 23 blue sharks (*Prionace glauca*) from the northern Tropical Eastern Pacific (TEP), a key corridor for global fisheries and debris transport associated with the North Pacific Garbage Patch. All individuals contained AMPs (mean ± SD = 32 ± 37 particles per digestive tract). Most particles were < 5 mm, with fibers as the dominant shape (76%). Among synthetic polymers, polyethylene (PE) and polyethylene terephthalate (PET) were the most abundant. Microplastics (MPs) showed a mean abundance of 12 ± 11 particles per digestive tract, whereas more than 60% of FTIR-confirmed particles corresponded to non-plastic anthropogenic microparticles (NPAMPs), mainly cotton, rayon, and cellulose, with a mean abundance of 22 ± 27 particles per digestive tract. Generalized additive models (GAMs) identified NPAMP abundance as the strongest predictor of THg concentrations (mean ± SD = 1.08 ± 0.43 mg kg⁻^1^), revealing a significant non-linear relationship with higher Hg levels during the hot–rainy season. These findings suggest that NPAMPs may represent, together with diet, an additional pathway associated with Hg exposure in *P. glauca*. Risk indices (PLI, MPDI, and PHI) indicated low-to-moderate contamination levels; however, more than half of the individuals exceeded polymer hazard thresholds (PHI > 1000). Given the observational design, limited sample size, and the fact that seasons were sampled in different years, the NPAMP–THg relationship should be interpreted as co-occurrence rather than direct evidence of contaminant transfer. Furthermore, because particles were quantified only in non-edible tissues, these findings cannot be directly extrapolated to seafood safety risk. Nevertheless, NPAMPs emerge as a previously under-recognized component of contaminant exposure in pelagic predators and should be incorporated into future marine monitoring frameworks.

**Supplementary Information:**

The online version contains supplementary material available at 10.1007/s11356-026-38018-x.

## Introduction

The widespread production and persistence of synthetic materials have generated a global pollution challenge. In aquatic environments, physical and chemical degradation processes transform larger debris into microparticles (< 5 mm), increasing their capacity to interact with other contaminants such as persistent organic pollutants and heavy metals, thereby enhancing their bioavailability to marine organisms (Liu et al. 2022; Koelmans et al. 2022). Pollution by anthropogenic microparticles (AMPs) arises not only from synthetic polymers such as plastics but also from anthropogenic modified natural fibers, including cotton and regenerated cellulose, largely originating from the textile industry (Wang et al. 2024). These materials, often treated with dyes and chemical additives, are not strictly classified as microplastics, yet they are environmentally persistent and increasingly recognized as emerging contaminants of concern (Acharya et al. 2021).

Microplastic (MP) ingestion by fish has been extensively documented (Lusher et al. 2013; Neves et al. 2015; de Vries et al. 2020), whereas non-plastic anthropogenic microparticles (NPAMPs) have only recently acquired attention in marine pollution research. Nevertheless, their ecological implications, particularly in top predators such as sharks, remain poorly understood (Savoca et al. 2021).

Sharks are especially relevant as sentinel species for marine pollution because of their high trophic position, broad distribution, and commercial importance (Riesgo et al. 2023). Among them, the blue shark (*Prionace glauca*) dominates industrial and artisanal catches in the Tropical Eastern Pacific (TEP), a region where floating debris associated with the North Pacific Garbage Patch converges with major fishing grounds (Lebreton et al. 2024; Egger et al. 2020). The TEP is also an important source of seafood for local and international markets, raising concerns about contaminant transfer through marine food webs (Rechimont et al. 2025).

Microplastics are widely recognized for their capacity to adsorb and transport heavy metals such as mercury (Hg) in aquatic environments (Yu et al. 2019; Liu et al. 2022; Wang et al. 2024). Recent experimental studies have further demonstrated that natural and modified cellulose-based microparticles can also exhibit high affinity for Hg through mechanisms including surface complexation and ion exchange (Sánchez-Moreno et al. 2025). Given the increasing abundance of NPAMPs, particularly semi-synthetic and processed textile fibers released from domestic laundry effluents, these materials may also contribute to Hg transport within marine food webs (Santonicola et al. 2025). However, demonstrating a mechanistic relationship between AMPs and heavy metals in marine megafauna remains challenging because of the complexity of trophic interactions and multiple environmental exposure pathways.

Based on this context, the objectives of this study were to (1) quantify and characterize anthropogenic microparticles ingested by blue sharks in the northern TEP; (2) evaluate whether AMP abundance in the gastrointestinal tract was associated with Hg concentrations in liver and muscle tissues; and (3) assess potential ecological and health risks using pollution load and polymer hazard indices. We hypothesized that AMPs and Hg would co-occur in *Prionace glauca* because both contaminants share environmental sources and transport pathways in the TEP, with their abundance varying jointly across seasons.

## Materials and methods

### Study area and sample preparation

Blue shark (*Prionace glauca*) specimens were obtained from two sampling periods in the northern Tropical Eastern Pacific (TEP). Ten individuals were collected during an oceanographic cruise conducted by the Mexican Institute for Sustainable Fisheries and Aquaculture Research (IMIPAS) in July 2019 (hot–rainy season) in the southern Gulf of California, whereas 13 individuals were obtained from commercial fishing vessels arriving at Mazatlán Harbor, Sinaloa, in January 2021 (cold–dry season), totaling 23 individuals.

**Fig. 1:**
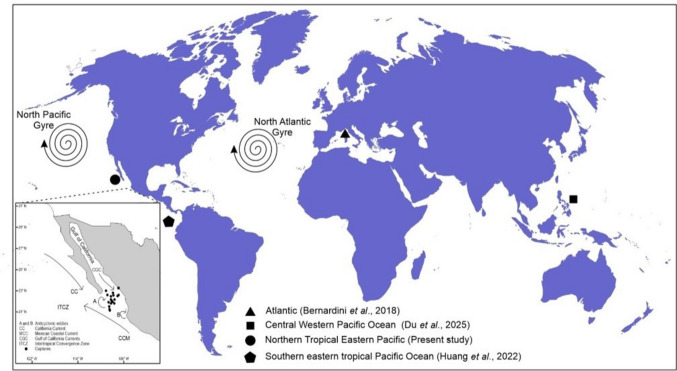
Study area and capture locations of blue sharks sampled in the northern TEP

The study area is influenced by the interaction between the North Equatorial Current and the California Current and represents an important transport pathway for floating debris associated with the North Pacific Garbage Patch into the eastern Pacific boundary current system (Fig. 1).

All sharks were captured using pelagic longline gear deployed from the surface to approximately 600 m depth during nighttime operations. Fishing gear consisted of nylon monofilament lines (~ 4 mm diameter) extending up to 55.6 km and equipped with approximately 200 circular hooks (16/0).

Biometric measurements, including total length (TL, cm), total weight (TW, kg), and sex, were recorded for each individual (Table S2). Specimens were classified into maturity stages according to criteria established for the species (Blanco-Parra 2003). Digestive tracts (stomach and intestine) were carefully dissected using clamps to prevent content loss and subsequently processed for anthropogenic microparticle analysis.

### Particle extraction process

All dissections and sample processing were conducted under controlled laboratory conditions to minimize airborne and procedural contamination. Each digestive tract was visually inspected using a magnifying lens to detect the presence of macroplastics (> 5 mm). After confirming their absence, stomach and intestine samples were transferred to pre-cleaned 800 mL glass containers.

Organic matter was removed using an alkaline–oxidative digestion protocol adapted from previously published methodologies for anthropogenic microparticle (AMP) isolation (Cole et al. 2014; Bessa et al. 2019; Yu et al. 2019), with modifications to optimize digestion efficiency for elasmobranch tissues. The digestion solution consisted of 20% KOH (m/v) and 30% H₂O₂ (v/v) mixed in a 3:1 ratio.

To assess method efficiency, recovery tests were performed using polyethylene microparticles of known origin (Cospheric LLC 2025) provided through the EPHEMARE framework (JPI Oceans 2025). Twenty particles (45–53 µm; Fig. S3), previously measured and documented, were added to each sample before digestion to evaluate particle recovery throughout the procedure.

Following digestion, samples were vacuum-filtered through Whatman® GF/D glass microfiber filters (2.7 µm pore size). Filters were dried at room temperature (25 ± 5 °C) and stored in covered glass Petri dishes until analysis.

### Microparticle identification and characterization

Filters were examined under a stereomicroscope (Carl Zeiss Microscopy 508 GmbH, Jena, Germany) equipped with a digital imaging system (AxioCam ERC 5 s digital camera). Suspected anthropogenic microparticles were identified based on morphological characteristics, including shape, color, and structure, following established guidelines (Lusher et al. 2020). Particle size was determined using ZEN 2 (Blue Edition) software. Suspected particles were subsequently isolated onto new glass fiber filters for chemical characterization.

Because of the high number of recovered particles, approximately 50% of the visually identified particles from each filter were randomly selected for Fourier transform infrared spectroscopy (FTIR) analysis. To minimize selection bias, each filter was visually divided into equal quadrants, and particles were selected across all quadrants using a random spatial approach rather than targeting specific morphologies, colors, or sizes. Selection was therefore stratified at the sample level rather than by particle morphology, ensuring representative coverage of the entire filter surface (Lusher et al. 2020).

FTIR analyses were performed using a Thermo Scientific Nicolet iN10 microscope. Spectra were acquired between 650 and 4000 cm⁻^1^ with a resolution of 4 cm⁻^1^. Polymer identification was achieved through comparison with reference spectral libraries, accepting only matches above 75% similarity (Thermo Fisher Scientific Inc. 2008a, b; Homemade spectral Library 2024). Particles were classified into two main categories: (1) microplastics (MPs), corresponding to synthetic polymers, and (2) non-plastic anthropogenic microparticles (NPAMPs), including cotton, cellulose, and rayon fibers. Particles identified as non-anthropogenic were excluded from further analyses.

### Contamination control

Strict contamination mitigation protocols were implemented throughout all analytical procedures. Procedural blanks (negative controls) were included during dissection, digestion, filtration, and microscopic observation. Negative controls consisted of Petri dishes containing filtered Milli-Q water exposed within the working area and processed alongside samples.

All laboratory procedures were conducted using non-plastic materials (glass or metal), and personnel wore cotton laboratory coats and nitrile gloves. Equipment and containers were covered with aluminum foil to minimize airborne contamination. Particles detected in procedural blanks were characterized and subsequently subtracted from sample counts based on matching morphological characteristics.

### Microplastics risk assessment

Ecological risk associated with anthropogenic microparticles was evaluated using three complementary indices: pollution load index (PLI), microplastic diversity index (MPDI), and polymer hazard index (PHI).

The PLI was calculated following Huang et al. (2022) as

$$PLI=\sqrt{\left(C_i/C_0\right)}$$

Where Ci represents the individual microplastic abundance, and C_0_ corresponds to a baseline reference value. Because of the absence of regional baseline data, the minimum abundance previously reported for blue sharks (11.21 items individual⁻^1^; Du et al. 2025) was used as the reference value.

The MPDI was calculated using a modified Simpson diversity index incorporating particle size, shape, color, and polymer type (Li et al. 2021; Wang et al. 2024).

The PHI was estimated following Lithner et al. (2011), considering both the relative abundance of each polymer type and its associated hazard score and thereby reflecting the potential toxicological risk of ingested particles (Table S4).

### Statistical analysis

Total mercury concentrations (THg) were calculated as the sum of liver and muscle Hg concentrations previously estimated for the same individuals in a prior study (Rechimont et al. 2024). Numerical variables were initially inspected using histograms and box plots, and normality was evaluated using Shapiro–Wilk tests. Because MPs, NPAMPs, and MPTA showed non-normal right-skewed distributions, non-parametric analyses were applied for group comparisons. THg showed a positive continuous distribution; therefore, generalized additive models (GAMs) with a Gamma error distribution and log link function were selected to account for positive responses and potential heteroscedasticity.

Frequency of occurrence (FO%) of anthropogenic microparticles (AMPs) was calculated independently for each classification criterion (organ of origin, particle shape, and material type), as these categories represented different attributes of the same particle pool rather than mutually exclusive groups. In addition, stacked bar plots were used to visualize microparticle contributions according to organ (stomach vs intestine), particle shape (fiber vs fragment), and material type (MPs vs NPAMPs). Graphical visualizations were produced in R using the ggplot2 package (R Core Team 2025).

Differences in MPTA, MPs, NPAMPs, and THg among biological and environmental categories were evaluated using Kruskal–Wallis tests due to the non-normal distribution of most variables. Statistical analyses and graphical visualizations were conducted in R using the packages ggplot2 and patchwork (R Core Team 2025).

Candidate GAMs were constructed using different anthropogenic microparticle metrics, including total anthropogenic microparticle abundance (MPTA), microplastic abundance (MPs), and non-plastic anthropogenic microparticle abundance (NPAMPs), as explanatory variables for THg concentrations. Additional biological and environmental covariates, including sex, maturity stage, size class, total length, and season, were also evaluated. Alternative model structures included additive smooths and season-specific interaction smooths (e.g., s(NPAMPs, by = SEASON)). GAMs were fitted using the mgcv package in R (R Core Team 2025). Model complexity was controlled by restricting the basis dimension (*k* = 3) to reduce overfitting, given the limited sample size. Model selection was based on Akaike Information Criterion (AIC), deviance explained, adjusted *R*^2^, and diagnostic inspection using gam.check function. A summary of evaluated models is provided in Table S8.

## Results and discussion

### Quality assurance

Procedural contamination detected in laboratory blanks during quality assurance and quality control was low compared with that observed in specimens (Table 1S) and was consistent with levels reported in comparable studies (Munno et al. 2024). The number of particles recorded in blanks and controls (dissection, filtration, and visual inspection) was 118. From these, 48 were identified as anthropogenic particles by FTIR analysis (mean ± SD: 8 ± 6 particles). Non-plastic anthropogenic particles were characterized primarily by cellulose (41.7%) and cotton (20.8%), followed by polymethyl methacrylate (PMMA) (16.7%), urethane alkyd (10.4%), rayon (6.3%), and polyester (4.2%) (Fig. S2). The occurrence of these materials in blanks is expected and attributed to laboratory-derived contamination, particularly from airborne textile fibers and paint-related particles (Woodall et al. 2015; Hermsen et al. 2018). Recovery efficiency from the negative controls reached a total recovery percentage (100%) across all replicates, although minor changes in EPHEMARE particle morphology and apparent size were observed.

The digestion method proved effective for organic matter removal while generally preserving particle integrity, as previously reported for hydrogen peroxide-based digestion protocols in microplastic analyses (Nuelle et al. 2014). However, oxidative treatments may still affect certain polymer types, particularly polyamides, and may induce minor alterations in aged polyethylene particles (Pfohl et al. 2021; Savino et al. 2022).

Although contamination levels were substantially lower than those observed in biological samples, a blank correction was applied to samples to account for procedural contamination and avoid overestimation of microparticle abundance, following recommended guidelines (Noonan et al. 2023).

### Anthropogenic microparticles: characterization and abundance

A total of 3039 suspected anthropogenic microparticles were visually identified across all digestive tract samples (23 stomachs and 23 intestines). Of these, 1443 particles (47.5%) were analyzed by FTIR, confirming 289 particles (~ 20%) as synthetic polymers (microplastics, MPs) and 443 particles (~ 30%) as non-plastic anthropogenic microparticles (NPAMPs). The remaining particles (~ 50%) were identified as non-anthropogenic material and excluded from further analysis.

**Fig. 2:**
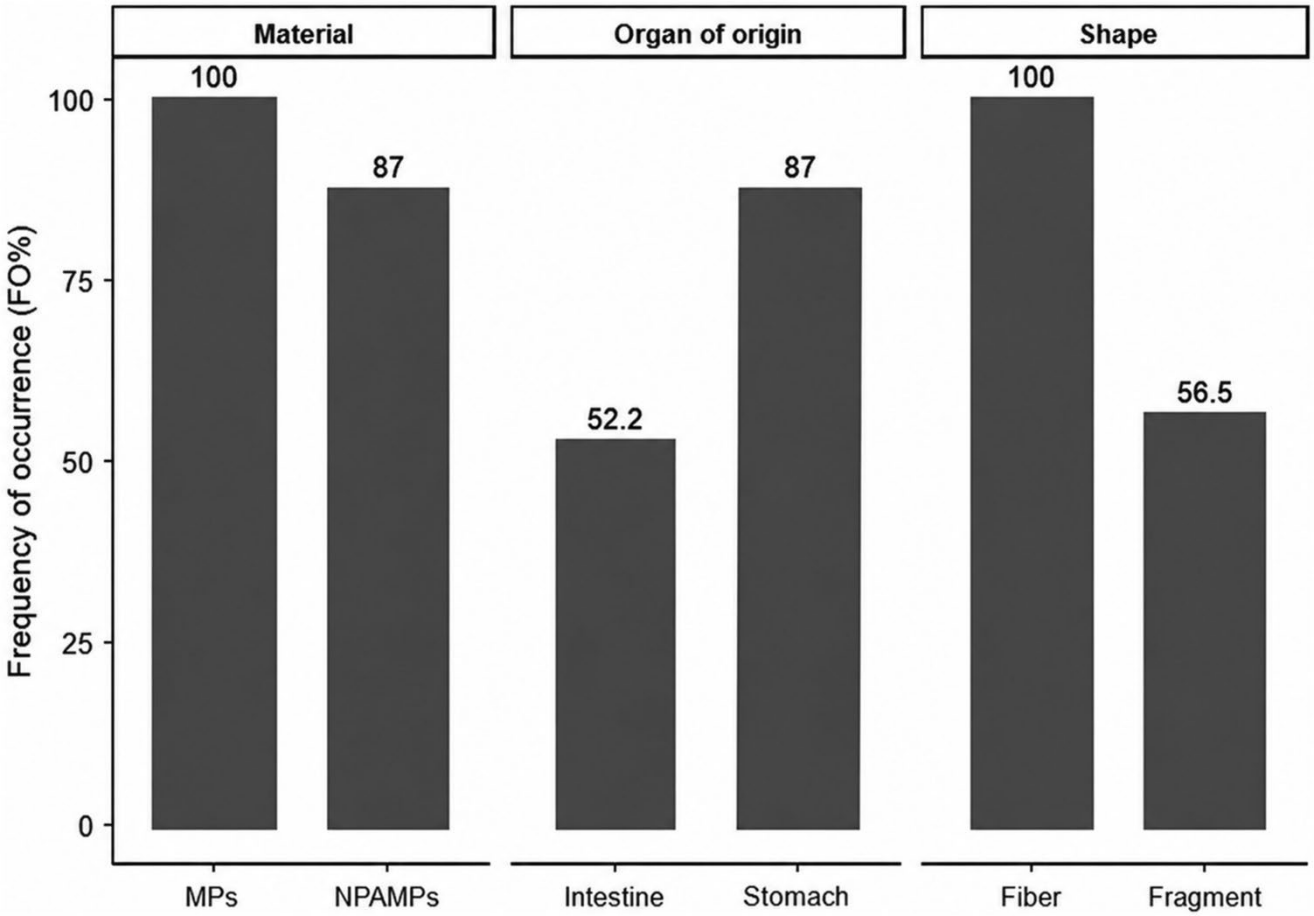
Frequency of occurrence (FO%) of AMPs in the gastrointestinal tract of *Prionace glauca* by organ (stomach vs intestine), shape (fiber vs fragment), and material type (MPs vs NPAMPs)

All blue shark specimens contained at least one anthropogenic microparticle, resulting in a frequency of occurrence (FO%) of 100% for anthropogenic microparticles (AMPs). Mean total abundance was 32 ± 37 microparticles per digestive tract (Table S2). MPs were present in 100% of individuals, whereas NPAMPs were detected in 87% of specimens. The stomach showed a higher frequency of occurrence (87%) than the intestine (56.5%), suggesting differential retention or ingestion patterns. Fibers were the dominant particle shape (100% occurrence), whereas fragments were less frequent (30%) (Fig. 2). Most particles were < 5 mm, ranging from 0.002 to 7.5 mm in length (mean ± SD: 0.7 ± 0.4 mm).

**Fig. 3:**
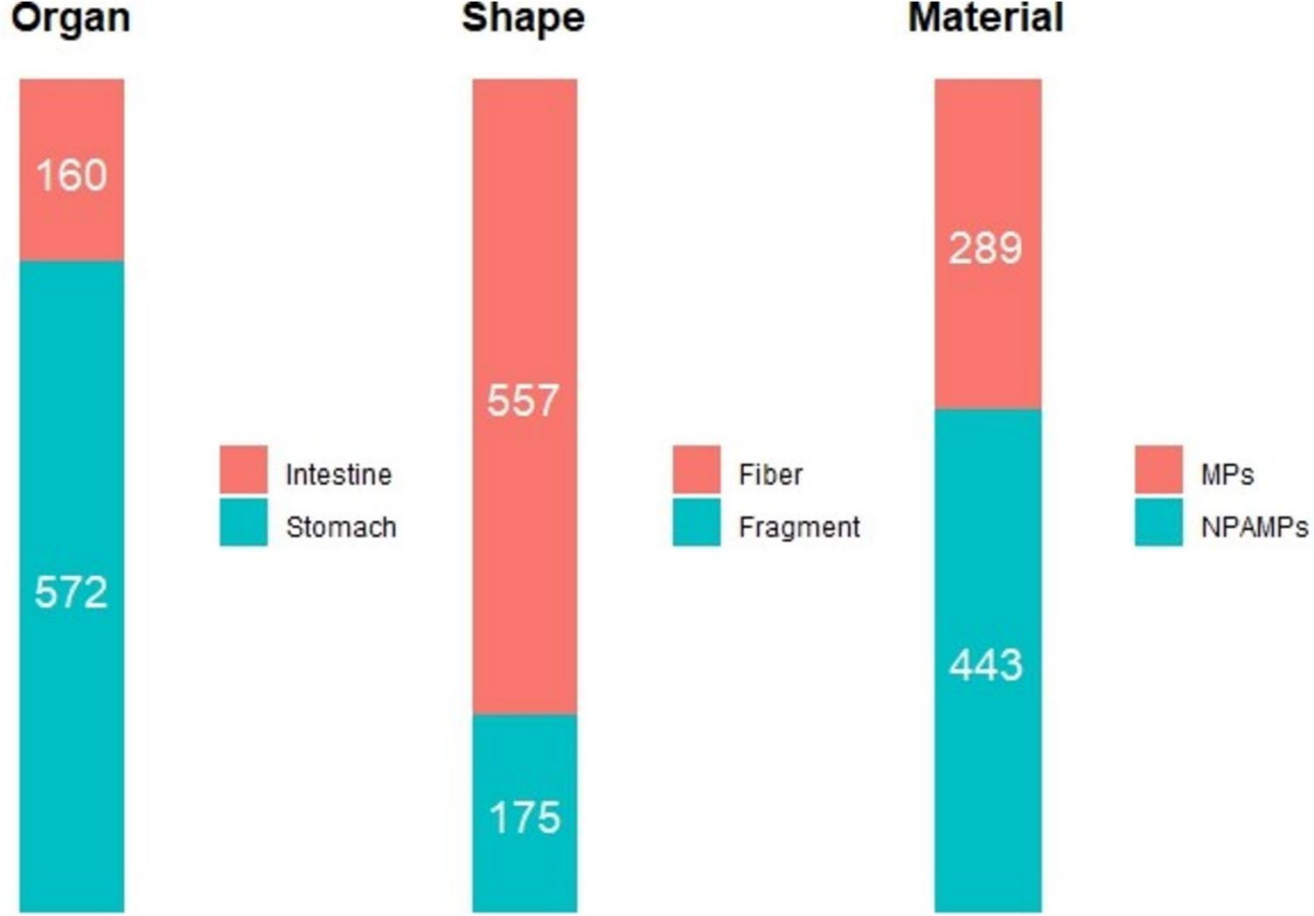
Anthropogenic microparticle abundance found in *Prionace glauca* gastrointestinal tract, by organ, shape, and material type (plastic and non-plastic). Values indicate counts of total particles isolated

Nine color categories were identified, with black (60%) and blue (30%) being the most common. FTIR analysis identified 11 polymer types, with polyethylene (PE), polyethylene terephthalate (PET), polystyrene (PS), and acrylic (ACRY) as the dominant synthetic polymers. Among NPAMPs, cotton, cellulose, and rayon dominated the composition. Anthropogenic microparticle abundance and composition observed in *Prionace glauca* were consistent with patterns reported for other elasmobranchs, summarized in Table 1, particularly the predominance of fibers and PE/PET polymers across pelagic predators. Similar fiber-dominated patterns have been reported in tiger sharks, hammerhead sharks, dolphins, and sea turtles, where fibers and textile-derived particles are consistently among the most abundant materials detected (Malthaner et al. 2024; Munno et al. 2024; Aierken et al. 2024). In the present study, NPAMPs exceeded MPs in abundance, reinforcing the growing recognition of textile-derived fibers as a major and still underrecognized component of marine pollution (Fig. 3). The predominance of fibers likely reflects their high environmental availability, buoyancy, and continuous release from domestic and industrial wastewater sources (Barrows et al. 2018). Likewise, the dominance of PE and PET is consistent with their widespread global production, durability, and resistance to degradation, which enhance their persistence and probability of ingestion by pelagic organisms (PlasticsEurope 2023).

**Table 1:** Summary of anthropogenic particle contamination reported in sharks from different marine regions. Information includes particle shape, size category, frequency of occurrence, abundance, and dominant polymer/material when available. Size categories: micro- (< 5 mm), meso- (5–25 mm), and macroplastics (> 25 mm). Abundance is expressed as particles per organ or per gram wet weight (ww), according to each study

Species	Main shape	Size range	Incidence %	Anthropogenic particle abundance	Most common polymer	Region	Reference
*Prionace glauca*	Sheet, fragment	Micro, meso, macro	25.26%	1–30 particles/stomach	PE	Mediterranean (Italy)	Bernardini et al. (2018)
*Prionace glauca*	Strings	Macro	1.18%	Entanglement	Fishing nets	South Pacific (Chile)	Mucientes and Queiroz (2019)
*Prionace glauca*	Sheet, fragment	Macro	2.2%	0.02 ± 0.1 particles/stomach	PE	South Pacific (Peru)	Fernández and Anastasopoulou (2019)
*Prionace glauca*	Sheet, fragment	Macro	25%	Macroplastic pieces (entanglement)	Plastic bag (PE) hard material boot	South Atlantic (Brazil)	Barreto et al. (2019)
Demersal Sharks	Fibers	Micro	67%	4 ± 5 particles/digestive tract	Cellulose polyacrylamide	NE Atlantic (UK)	Parton et al. (2020)
*Lamna nasus*	Fragments	Micro	N/A	5.5 ± 2.7 particles/intestine	N/A	NE Atlantic (UK)	Maes et al. (2020)
*Scyliorhinus canicula*	Fibers	Micro, meso, macro	80.3%	2.4 particles/digestive tract	N/A	Mediterranean (Italy)	Messinetti et al. (2022)
*Prionace glauca*	Fibers	Micro	39.1%	0.15 ± 0.38 items/g ww pyloric tissue	PET	Tropical Western Pacific (China)	Huang et al. (2022)
*Isurus paucus*	Plastic bag Plastic bottle	Macro	100%	N/A	Polypropylene	Tropical Western Pacific (China)	Gong et al. (2023)
*Rhizoprionodon porosus*	Fibers	Micro	100%	13.8 particles/digestive tract	PE	South Atlantic (Brazil)	Melo et al. (2024)
Multiple species	Fibers	Micro	100%	2–31 particles/digestive tract	Rayon	Tropical Western Pacific (Taiwan)	Wang et al. (2024)
*Sphyrna lewini*	Fibers	Micro	79.3%	N/A	Cellulose	Gulf of California (Mexico)	Malthaner et al. (2024)
*Galeocerdo cuvier*	Fragment fibers	Micro	NA	450 ± 513 particles/stomach	Polypropylene cellulose	US Atlantic and Gulf of Mexico	Munno et al. (2024)
*Carcharhinus macloti/Maculabatis gerrardi*	Fibers	Micro	100%	12.6 particles/g tissue (muscle, gills, and stomach)	Polycarbonate	Persian Gulf and Sea of Oman	Pasalari et al. (2025)
*Prionace glauca*	Fibers	Micro	95.45%	11.21 ± 9.62 (particles/intestine)	Polyester	Tropical Western Pacific (China)	Du et al. (2025)
*Prionace glauca*	Fibers	Micro	100%	32 ± 37 particles/digestive tract	PE cellulose	Tropical Eastern Pacific (Mexico)	Present study

No significant differences in anthropogenic microparticle total abundance (MPTA) were detected among sex, maturity stage, season, or size classes (Kruskal–Wallis tests, *p* > 0.05 in all cases) (Table S6), suggesting that exposure is primarily driven by environmental availability rather than individual traits. In highly migratory pelagic species such as *Prionace glauca*, continuous exposure to microparticles distributed throughout the water column likely explains the observed uniformity (Choy et al. 2019; Egger et al. 2020).

### Risk assessment

Overall, risk indices indicated low-to-moderate exposure levels, although substantial variability was observed among individuals. PLI values remained below 10 for all specimens (mean ± SD: 1.18 ± 0.65) (Table S3), suggesting relatively low contamination levels. MPDI values were also low (mean ± SD: 0.31 ± 0.28), indicating limited diversity in particle characteristics (Table S4).

These values are consistent with previous studies reporting low contamination scenarios in marine organisms, although still ecologically relevant due to chronic multiple exposure pathways (Arivukumar et al. 2026).

In contrast, PHI values were markedly elevated (mean ± SD: 24,604 ± 34,778), with 56% of individuals exceeding the threshold of 1000 (Table S5), indicating high to extreme hazard levels. This discrepancy between abundance-based indices (PLI, MPDI) and hazard-based metrics (PHI) highlights that risk is not solely determined by particle quantity, but also by polymer composition and associated chemical toxicity (Lithner et al. 2011).

From a food safety perspective, these findings are particularly relevant, as even moderate ingestion levels may translate into elevated toxicological risk when high-hazard polymers are involved. This reinforces concerns about the potential co-exposure to other contaminants, such as heavy metals, through seafood consumption.

### Seasonal trends in anthropogenic microparticles and mercury co-occurrence

**Fig. 4:**
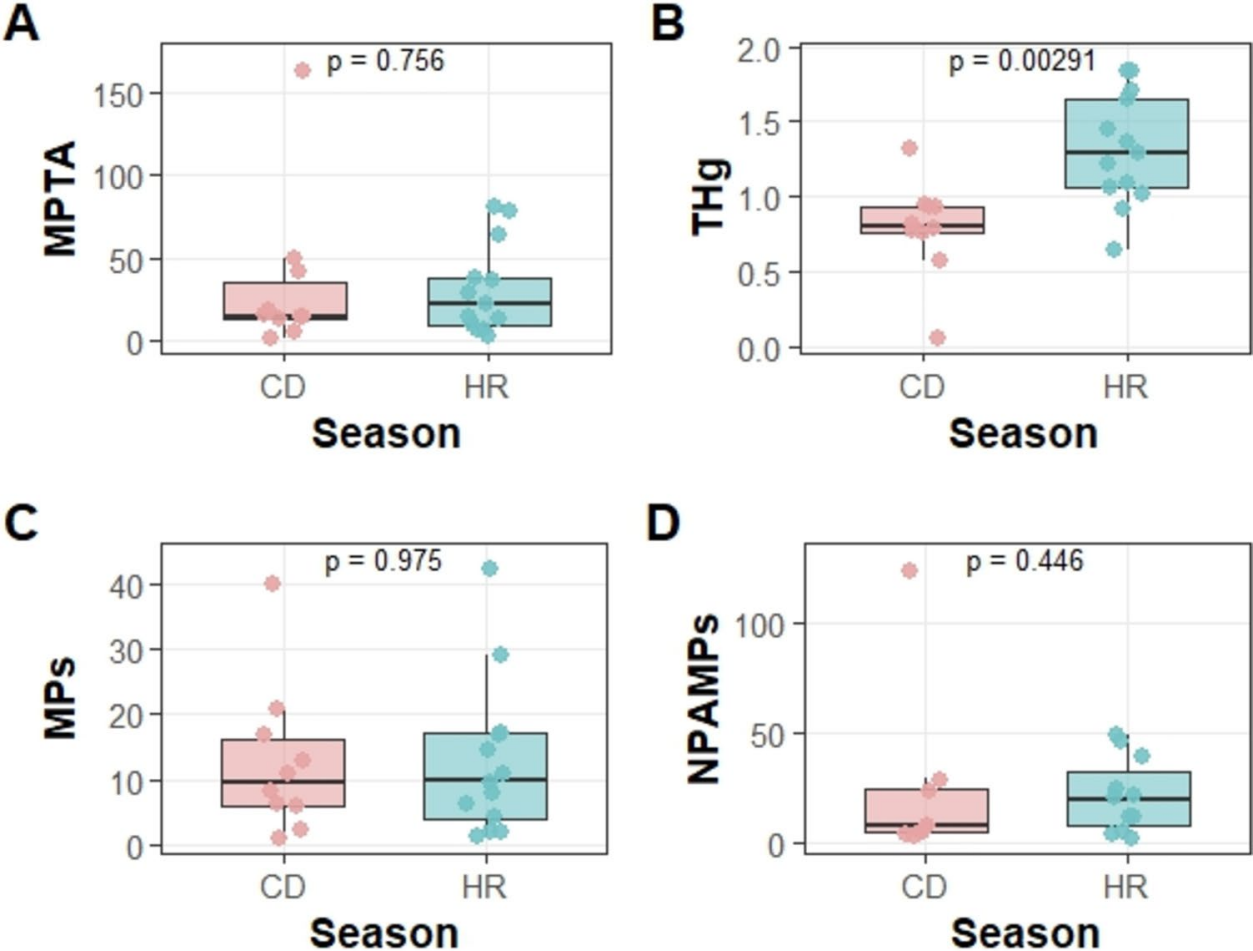
Seasonal variation in anthropogenic microparticles and total mercury concentrations (THg) in *Prionace glauca*. Boxplots compare the cold–dry (CD) and hot–rainy (HR) seasons for **A** total anthropogenic microparticles (MPTA), **B** THg, **C** microplastics (MPs), and (**D**) non-plastic anthropogenic microparticles (NPAMPs). Points represent individual sharks. *p*-values correspond to Kruskal–Wallis tests

**Fig. 5:**
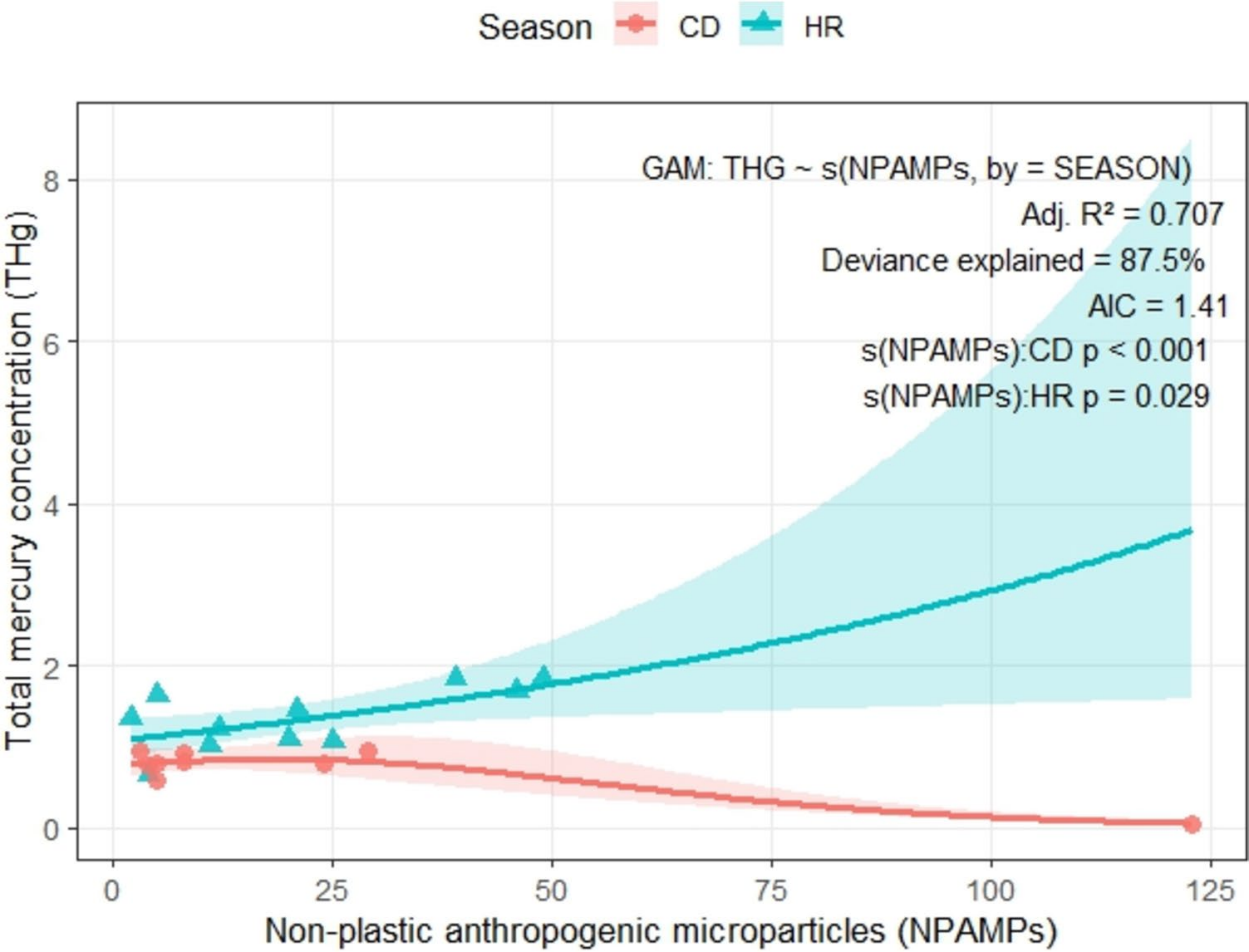
Generalized additive model (GAM) showing the relationship between total mercury concentrations (THg) and non-plastic anthropogenic microparticles (NPAMPs) in *Prionace glauca* across seasons. Lines represent season-specific smooths and shaded areas indicate 95% confidence intervals. Model statistics are shown within the panel

Mean THg concentrations in liver and muscle tissues reached 1.08 ± 0.43 mg kg⁻^1^ (mean ± SD; Table S6), with significantly higher values detected during the hot–rainy (HR) season compared with the cold–dry (CD) season (Kruskal–Wallis, *p* = 0.003; Fig. 4B). Anthropogenic microparticles total abundance (MPTA) showed a similar increasing tendency during the HR period; however, seasonal differences were not statistically significant (Fig. 4A). This trend appeared to be primarily associated with the greater abundance of non-plastic anthropogenic microparticles (NPAMPs) observed during the HR season (Fig. 4D), whereas microplastics (MPs) abundance remained relatively stable between seasons (Fig. 4C). Generalized additive models (GAMs) further indicated that the model including season-specific smooths for NPAMPs abundance provided the best overall fit for explaining THg concentrations (AIC = 1.41; deviance explained = 87.5%; adjusted *R*^2^ = 0.707), revealing significant NPAMP–THg relationships in both sampling periods but with different seasonal response patterns (Fig. 5). Increased contaminant levels during the HR season may reflect enhanced land-based inputs associated with rainfall, runoff, and hydrodynamic transport processes, which have been shown to increase the delivery of anthropogenic particles and associated contaminants into coastal marine ecosystems during rainy periods (Jayalath and Ratnayake 2025; Ríos-Mendoza et al. 2021; Saniewska et al. 2014). Together, these findings suggest that seasonal environmental dynamics may influence not only contaminant availability but also the co-occurrence patterns between NPAMPs and mercury in pelagic predators. Nevertheless, these temporal patterns should be interpreted cautiously because the two sampling periods corresponded to different years, preventing complete separation of seasonal and interannual environmental variability under the present study design.

Experimental studies have demonstrated that both microplastics and cellulosic-based anthropogenic fibers can adsorb Hg from aquatic environments, supporting their potential role as contaminant vectors (Gao et al. 2023; Zhou et al. 2024; Frost et al. 2024). Likewise, modified and semi-synthetic cellulosic fibers have shown strong Hg-binding capacities, suggesting that processed NPAMPs commonly detected in marine organisms may contribute to Hg transport and retention (Yoo et al. 2023; Algieri et al. 2024). Recent evidence further highlights that top predators and threatened marine species may be particularly vulnerable to the combined effects of anthropogenic particles and heavy metals because of contaminant bioaccumulation and biomagnification across trophic webs (Azhar and Khalid 2026). However, field-based evidence directly linking anthropogenic microparticles and Hg in free-ranging marine organisms remains extremely limited, particularly in elasmobranchs. Therefore, the positive relationship observed here between NPAMPs and THg should be interpreted as first evidence of co-occurrence rather than direct mechanistic transfer, since shared environmental sources, trophic pathways, and seasonal variability may also influence contaminant exposure.

### Implications for management and policy

Although anthropogenic microparticles (AMPs) were quantified only in the gastrointestinal tract, which is not typically consumed by humans, increasing evidence suggests that these particles can translocate to other tissues, including muscle and liver. Several studies have reported the presence of AMPs in edible fish tissues, indicating their ability to cross biological barriers and enter systemic circulation, especially those on the nanoparticle range (McIlwraith et al. 2021; Guerrera et al. 2021; Rochman and Hoellein 2020). However, the potential health risks associated with human ingestion of AMPs remain inconclusive. Current evidence is largely limited to mechanistic plausibility, experimental effects observed under relatively high exposure conditions, and signals of potential concern, whereas direct evidence in humans is still insufficient (WHO 2022).

In contrast, mercury follows a well-established toxicokinetic pathway, being primarily absorbed as methylmercury through diet and accumulating in protein-rich tissues such as muscle, with seafood representing one of the main routes of human exposure (Thomsen et al. 2022). Consequently, maximum permissible Hg concentrations in seafood have been established by national and international regulations, including NOM-129-SSA1-1995 (1995) and the U.S. Food and Drug Administration (FDA 2024). The toxicological effects of Hg exposure, particularly neurotoxicity and oxidative stress, are also well documented (Basu et al. 2023).

The potential interaction between AMPs and Hg suggests possible combined exposure pathways, in which anthropogenic particles may act as carriers or proxies of contamination sources. From a management and policy perspective, these findings highlight the importance of moving beyond gut-based assessments to include tissue-level analyses, while also considering interactions between emerging pollutants such as NPAMPs and established contaminants like mercury. Integrating these factors into seafood safety and marine monitoring frameworks may improve future risk assessments in marine food systems.

## Conclusions

Blue sharks (*Prionace glauca*) from the northern Tropical Eastern Pacific (TEP) were consistently exposed to anthropogenic microparticles, confirming that highly migratory pelagic predators integrate contamination across broad oceanic scales. Non-plastic anthropogenic microparticles (NPAMPs), particularly textile-derived fibers such as cotton, cellulose, and rayon, were more abundant than conventional microplastics (MPs), reinforcing the growing recognition of these materials as a major yet often overlooked component of marine pollution.

Among the variables evaluated, NPAMP abundance was the strongest predictor of total mercury concentrations (THg), showing a significant non-linear relationship with clear seasonal amplification during the hot–rainy period. However, this association should be interpreted cautiously as evidence of co-occurrence and shared exposure pathways rather than direct proof of mercury transfer mediated by anthropogenic particles. Alternative explanations, including overlapping contamination sources, trophic ecology, habitat use, and temporal variability in exposure, remain plausible.

Although abundance-based indices suggested relatively low-to-moderate contamination levels, the elevated polymer hazard index (PHI) observed in several individuals indicates that ecological risk depends not only on particle quantity but also on polymer composition and associated chemical toxicity. This distinction is particularly relevant for sharks, which are commercially important and already recognized for their tendency to accumulate mercury through biomagnification.

Despite the limited sample size, this study provides novel baseline evidence for a commercially important apex predator and contributes to the emerging framework addressing interactions between anthropogenic microparticles and chemical contaminants. The results further highlight the need to incorporate NPAMPs into marine monitoring and risk assessment strategies. Future research should experimentally evaluate AMP–mercury interactions through controlled exposure studies and tissue-level analyses, particularly in edible tissues, to better understand their potential implications for seafood safety and contaminant transfer within marine food webs.

## Supplementary Information

Below is the link to the electronic supplementary material.

Supplementary Material File 1 (539 KB)

## Data Availability

The datasets are available upon reasonable request.
