# Supplementary material for: Anthropogenic microparticles and mercury co-occurrence in blue sharks from the Tropical Eastern Pacific
Source: Environ Sci Pollut Res Int. 2026 Jul 4;33(21):10745–56. doi: 10.1007/s11356-026-38018-x (PMC13368941; doi:10.1007/s11356-026-38018-x)
Supplement: Supplementary file 1 — Supplementary Material File 1 (539 KB) [file 11356_2026_38018_MOESM1_ESM.docx]

**Supplementary material**


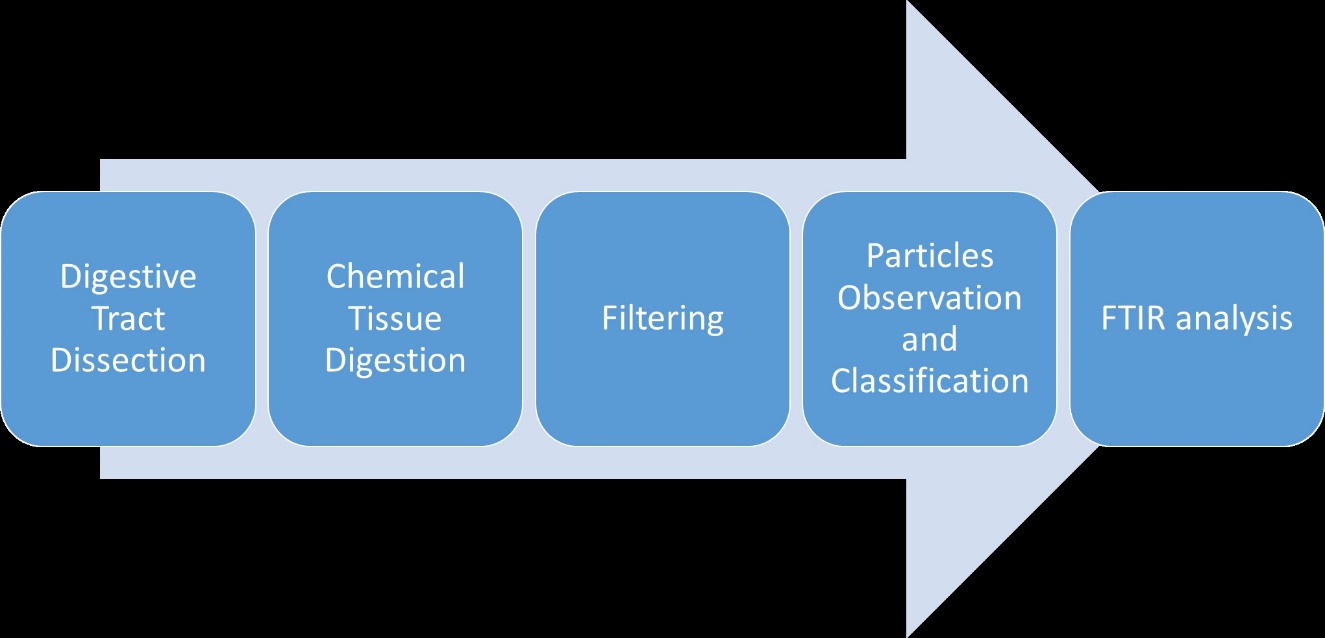


**Fig.S1.** Diagram of microplastics isolation, quantification, and characterization: flux of work.

**Table S1.** Composition of the particles isolated from quality controls.

| **Polymer type** | **Total particles** |
| --- | --- |
| Cellulose (CE) | 20 |
| Cotton (CO) | 10 |
| Polymethylmethacrylate (PMM) | 8 |
| Uretane Alkyd (UA) | 5 |
| Rayon (RA) | 3 |
| Polyester (PO) | 2 |
|  |  |

**
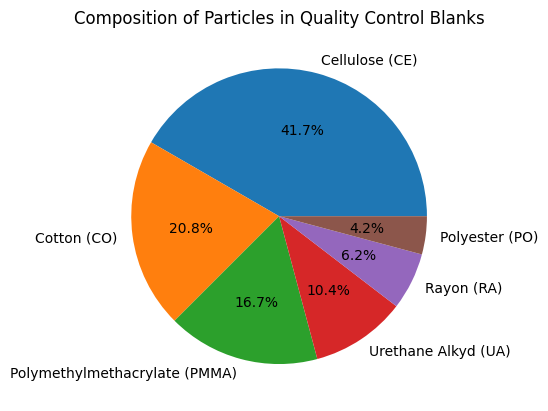
**

**Fig.S2:** Relative percentage composition of polymer types identified in quality control blanks.

**
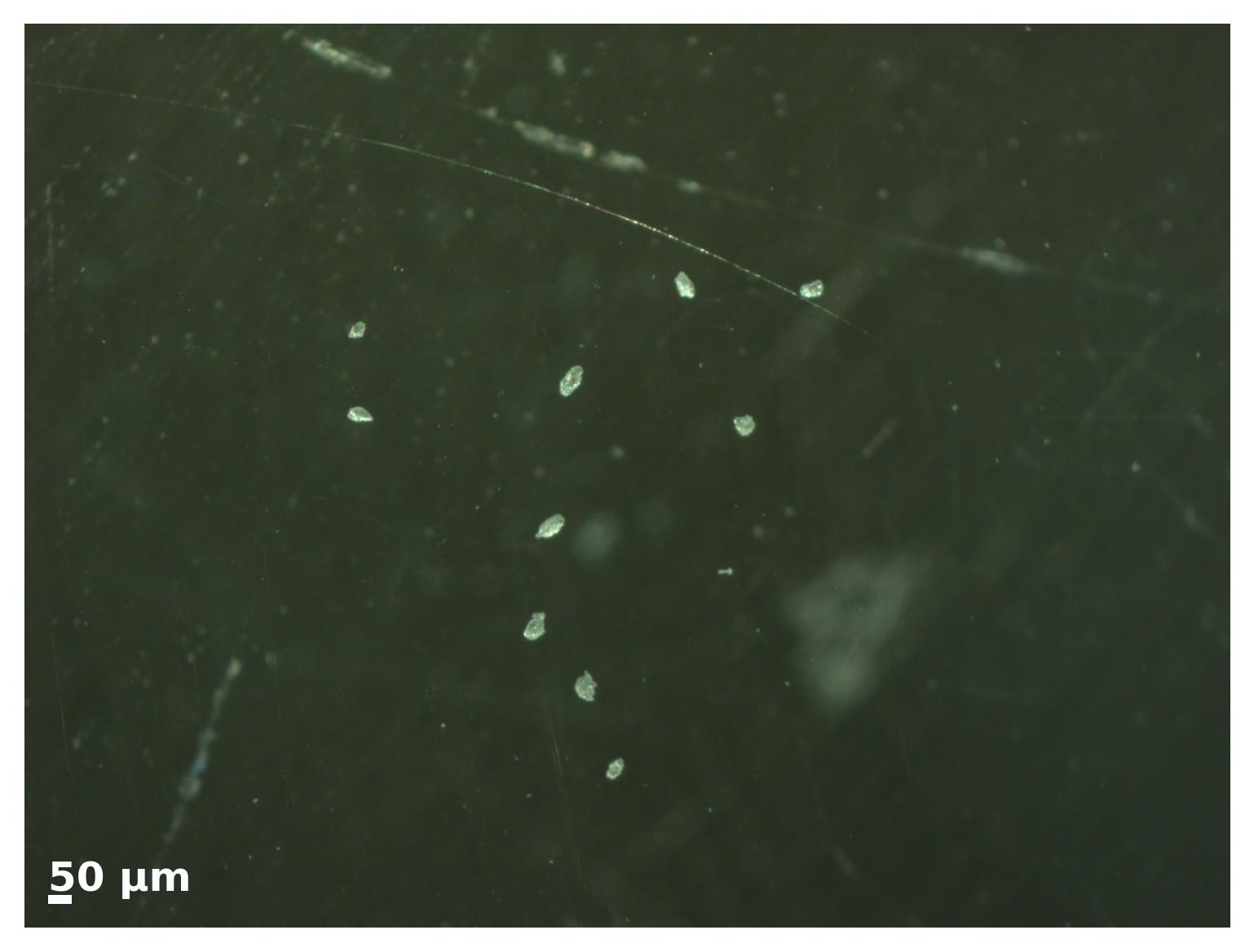
**

**Fig. S3.** Ephemeral irregular particles used as a positive control in sample digestion and filtration treatment.

**Table S2**. Biometric data of each organism (total length, sex, and stage) and anthropogenic microparticles abundance (AMPs) in the stomach (S), intestine (I), and in total (AMPTA).

|  | TL | SEX | STAGE | AMPS | AMPI | AMPTA |
| --- | --- | --- | --- | --- | --- | --- |
| PP1 | 164.4 | F | J | 3 | 0 | 3 |
| PP2 | 199.3 | M | A | 28 | 0 | 28 |
| PP3 | 181.6 | M | A | 6 | 3 | 9 |
| PP4 | 190 | M | A | 63 | 0 | 63 |
| PP5 | 241.8 | M | A | 0 | 38 | 38 |
| PP6 | 210.1 | M | A | 78 | 0 | 78 |
| PP7 | 214.4 | M | A | 52 | 29 | 81 |
| PP8 | 151.8 | F | J | 36 | 0 | 36 |
| PP9 | 166.6 | F | J | 13 | 2 | 15 |
| PP10 | 190.4 | M | A | 22 | 0 | 22 |
| PP11 | 211.5 | F | A | 4 | 2 | 6 |
| PP12 | 223.5 | M | A | 6 | 0 | 6 |
| PP13 | 202 | M | A | 13 | 0 | 13 |
| PP14 | 184 | M | J | 40 | 10 | 50 |
| PP15 | 154 | M | J | 5 | 36 | 41 |
| PP16 | 171 | M | J | 0 | 14 | 14 |
| PP17 | 183.5 | F | A | 5 | 9 | 14 |
| PP18 | 148 | M | J | 1 | 0 | 1 |
| PP19 | 191 | F | A | 8 | 5 | 13 |
| PP20 | 174 | M | J | 13 | 5 | 18 |
| PP21 | 219.5 | M | A | 15 | 0 | 15 |
| PP22 | 167.3 | M | J | 0 | 5 | 5 |
| PP23 | 193 | M | A | 163 | 0 | 163 |


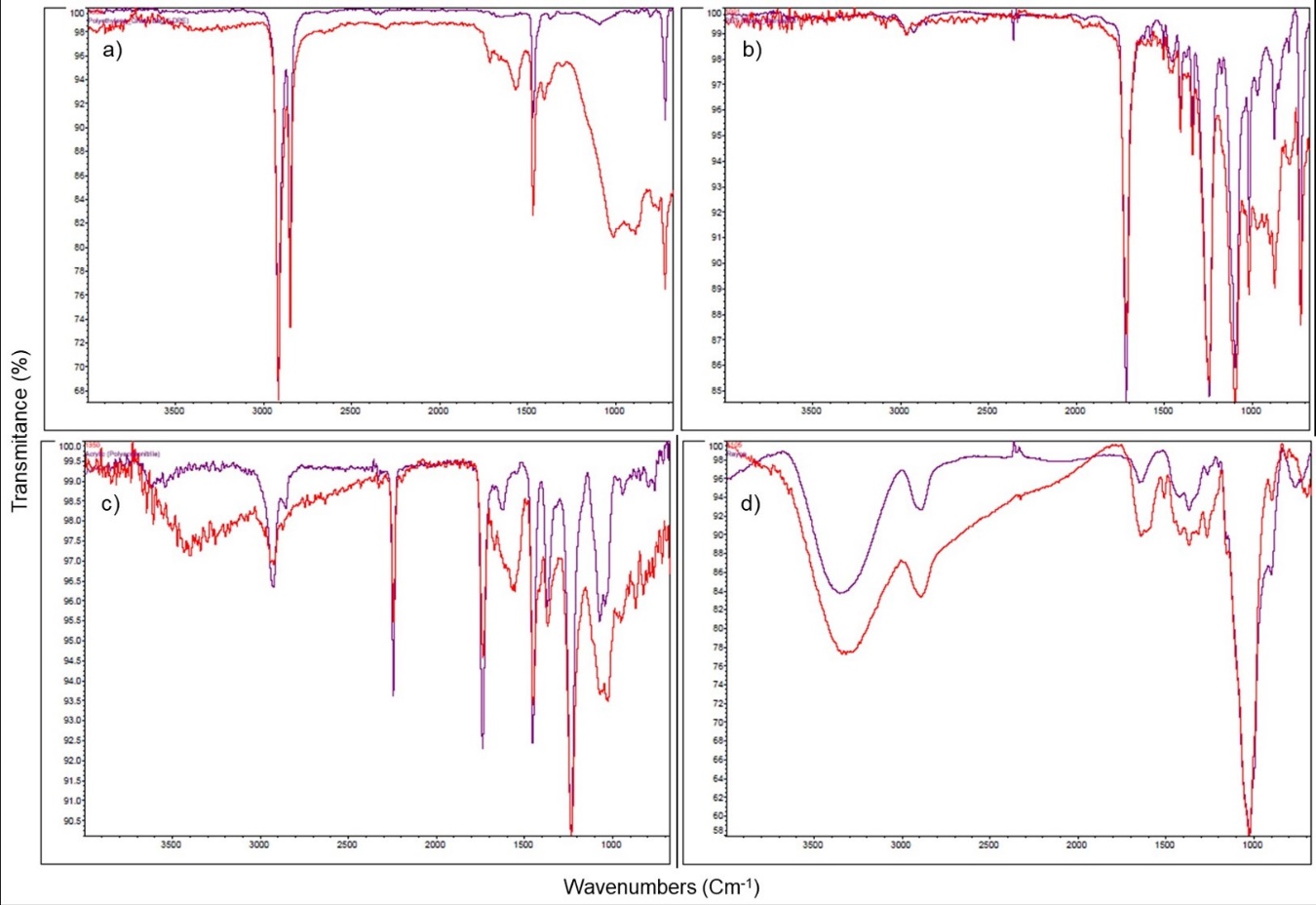


**Fig. S4.** Spectra of some of the polymers found in the samples by FTIR analysis: a) Polyethylene (96% match), b) Polyethylene Terephthalate (91%match), c) Acrylic (87% match) and d) Rayon (91% match).

**Table S3**. Microplastic Diverse Index (MPDI) and PLI (Pollution Load Index) for each blue shark organism captured.

|  | color | shape | size | polymer type | MPDI | PLI |
| --- | --- | --- | --- | --- | --- | --- |
| PP1 | 0.98 | 0.75 | 0.89 | 0.99 | 0.65 | 0.22 |
| PP2 | 0.94 | 0.75 | 0.89 | 0.85 | 0.53 | 0.63 |
| PP3 | 0.98 | 0.75 | 0 | 0.98 | 0 | 0.45 |
| PP4 | 0.61 | 0 | 0 | 0.85 | 0 | 0.92 |
| PP5 | 0.86 | 0 | 0 | 0.85 | 0 | 0.92 |
| PP6 | 0.61 | 0 | 0.56 | 0.79 | 0 | 1.20 |
| PP7 | 0.86 | 0 | 0.89 | 0.52 | 0 | 1.45 |
| PP8 | 0.98 | 0 | 0.00 | 0.91 | 0 | 0.74 |
| PP9 | 0.75 | 0 | 0.56 | 0.85 | 0 | 0.87 |
| PP10 | 0.98 | 0 | 0.56 | 0.79 | 0 | 0.71 |
| PP11 | 0.86 | 0.75 | 0.56 | 0.91 | 0.33 | 0.55 |
| PP12 | 0.98 | 0.75 | 0.56 | 0.98 | 0.41 | 0.32 |
| PP13 | 0.94 | 0.02 | 0 | 0.98 | 0 | 0.32 |
| PP14 | 0.98 | 0.75 | 0 | 0.71 | 0 | 1.02 |
| PP15 | 0.86 | 0.00 | 0.56 | 0.85 | 0 | 0.92 |
| PP16 | 0.94 | 0.75 | 0.56 | 0.91 | 0.36 | 0.55 |
| PP17 | 0.86 | 0.75 | 0.89 | 0.91 | 0.52 | 0.55 |
| PP18 | 0.98 | 0.75 | 0.56 | 0.99 | 0.41 | 0.22 |
| PP19 | 0.86 | 0.75 | 0.89 | 0.79 | 0.45 | 0.63 |
| PP20 | 0.86 | 0.00 | 0 | 0.85 | 0 | 0.81 |
| PP21 | 0.75 | 0.02 | 0 | 0.91 | 0 | 0.74 |
| PP22 | 0.94 | 0.75 | 0.56 | 0.98 | 0.39 | 0.32 |
| PP23 | 0.86 | 0.02 | 0 | 0.62 | 0 | 1.41 |

**Table S4.** Hazard and risk categories for microplastics (MPs) pollution (Ranjani et al., 2021).

| **PHI** | **Hazard category** | **Risk category** |
| --- | --- | --- |
| 0–1 | I | Minor |
| 1–10 | II | Medium |
| 10–100 | III | High |
| 100–1000 | IV | Danger |
| >1000 | V | Extreme danger |

**Table S5.** The polymer hazard index was determined for each blue shark analyzed.

| ID | PHI |
| --- | --- |
| PP1 | 40 |
| PP2 | 110.909091 |
| PP3 | 100 |
| PP4 | 20808.9552 |
| PP5 | 76.8421053 |
| PP6 | 932.692308 |
| PP7 | 15469.6333 |
| PP8 | 14922.807 |
| PP9 | 133.333333 |
| PP10 | 30.7692308 |
| PP11 | 31622.7273 |
| PP12 | 38672.2222 |
| PP13 | 26757.6923 |
| PP14 | 87987.3563 |
| PP15 | 59.4594595 |
| PP16 | 46513.3333 |
| PP17 | 70.3703704 |
| PP18 | 22.2222222 |
| PP19 | 89880.6452 |
| PP20 | 16227.907 |
| PP21 | 128922.222 |
| PP22 | 38672.2222 |
| PP23 | 7863.77953 |

#
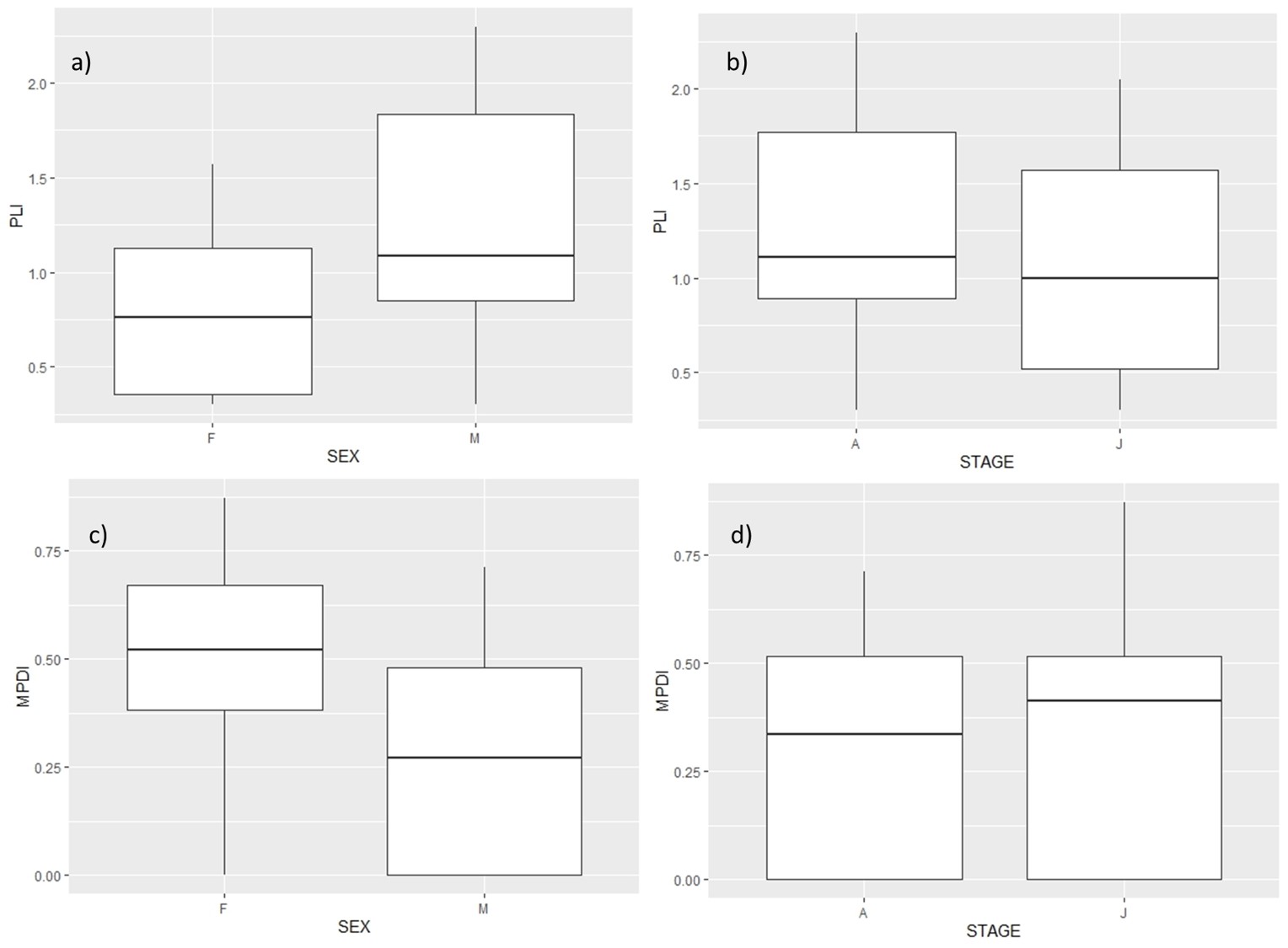


**Fig.S5.** PLI and MPDI mean and standard deviation discriminated by sex and stage (where F: Female, M: Male, A: Adult, J: Juvenile).

**Table S6.** Results of Kruskal–Wallis tests evaluating differences in total anthropogenic microparticle abundance (MPTA) among biological and environmental categories in *Prionace glauca* from the northern Tropical Eastern Pacific.

| **Variable** | **Factor** | **K-W H** | **df** | **p-value** |
| --- | --- | --- | --- | --- |
| MPTA | Sex | 2.17 | 1 | 0.141 |
| MPTA | Maturity stage | 0.895 | 1 | 0.344 |
| MPTA | Season | 0.096 | 1 | 0.756 |
| MPTA | Size class | 2.76 | 3 | 0.430 |

*No significant differences were detected among groups (p > 0.05).*

**Table S6.** Total mercury concentration in µg.g^-1^ (THg) found in each blue shark.

| ID | THg |
| --- | --- |
| PP1 | 1.363 |
| PP2 | 1.095 |
| PP3 | 1.645 |
| PP4 | 1.700 |
| PP5 | 1.453 |
| PP6 | 1.835 |
| PP7 | 1.834 |
| PP8 | 1.068 |
| PP9 | 0.922 |
| PP10 | 1.220 |
| PP11 | 1.287 |
| PP12 | 0.651 |
| PP13 | 1.015 |
| PP14 | 0.936 |
| PP15 | 0.781 |
| PP16 | 0.931 |
| PP17 | 0.818 |
| PP18 | 1.317 |
| PP19 | 0.577 |
| PP20 | 0.785 |
| PP21 | 0.762 |
| PP22 | 0.952 |
| PP23 | 1.134 |

**Table S7**. AMPs abundance calculated as the total number of anthropogenic microparticles found in each organ from the digestive tract by gram of wet weight from each tissue.

| ID | STOMACH | INTESTINE |
| --- | --- | --- |
| PP1 | 0.003 | 0.000 |
| PP2 | 0.027 | 0.000 |
| PP3 | 0.007 | 0.005 |
| PP4 | 0.035 | 0.000 |
| PP5 | 0.000 | 0.052 |
| PP6 | 0.090 | 0.000 |
| PP7 | 0.038 | 0.017 |
| PP8 | 0.039 | 0.000 |
| PP9 | 0.007 | 0.003 |
| PP10 | 0.034 | 0.000 |
| PP11 | 0.001 | 0.006 |
| PP12 | 0.006 | 0.000 |
| PP13 | 0.016 | 0.000 |
| PP14 | 0.036 | 0.006 |
| PP15 | 0.004 | 0.020 |
| PP16 | 0.000 | 0.025 |
| PP17 | 0.000 | 0.025 |
| PP18 | 0.002 | 0.000 |
| PP19 | 0.011 | 0.007 |
| PP20 | 0.010 | 0.010 |
| PP21 | 0.026 | 0.000 |
| PP22 | 0.000 | 0.004 |
| PP23 | 0.167 | 0.000 |

**Table S8.** Candidate generalized additive models (GAMs) evaluated to explain total mercury concentrations (THg) in *Prionace glauca*.

| **Model** | **Formula** | **AIC** | **ΔAIC** | **Deviance explained (%)** | **Adj. R²** | **Interpretation** |
| --- | --- | --- | --- | --- | --- | --- |
| NPAMPs_by_SEASON | THG ~ s(NPAMPs, by = SEASON) + SEASON | 1.41 | 0.00 | 87.5 | 0.707 | Selected final model |
| NPAMPs_SEASON_SIZE | THG ~ s(NPAMPs) + SEASON + SIZE | 2.88 | 1.47 | 89.5 | 0.689 | Similar fit but increased complexity |
| MPTA_by_SEASON | THG ~ s(MPTA, by = SEASON) + SEASON | 5.75 | 4.34 | 82.7 | 0.655 | Lower explanatory power |
| NPAMPs_SEASON | THG ~ s(NPAMPs) + SEASON | 6.95 | 5.54 | 81.7 | 0.518 | Simpler additive alternative |
| NPAMPs_SEASON_SEX | THG ~ s(NPAMPs) + SEASON + SEX | 8.79 | 7.38 | 82.1 | 0.497 | No improvement from SEX |
| NPAMPs_SEASON_TL | THG ~ s(NPAMPs) + SEASON + s(TL) | 9.00 | 7.59 | 81.9 | 0.474 | No improvement from TL |
| NPAMPs_SEASON_STAGE | THG ~ s(NPAMPs) + SEASON + STAGE | 9.10 | 7.70 | 81.8 | 0.474 | No improvement from STAGE |
| MPs_by_SEASON | THG ~ s(MPs, by = SEASON) + SEASON | 12.6 | 11.2 | 76.7 | 0.564 | MPs showed weaker relationships |
| NPAMPs | THG ~ s(NPAMPs) | 14.9 | 13.5 | 69.7 | 0.303 | Season improved model performance |
| MPTA_SEASON | THG ~ s(MPTA) + SEASON | 21.7 | 20.3 | 62.2 | 0.147 | Lower explanatory performance |
